# Six weeks of high‐intensity interval training improve autonomic and metabolic responses in active postmenopausal women

**DOI:** 10.1113/EP093739

**Published:** 2026-07-17

**Authors:** Jordi Monferrer‐Marín, Ainoa Roldán, Jørn Wulff Helge, Cristina Blasco‐Lafarga

**Affiliations:** ^1^ Sport Performance and Physical Fitness Research Group (UIRFIDE), Physical Education and Sports Department University of Valencia Valencia Spain; ^2^ Department of Biomedical Sciences, Faculty of Health and Medical Sciences University of Copenhagen Copenhagen Denmark

**Keywords:** autonomic nervous system, fatty acid oxidation, heart rate variability, high intensity interval training, oxygen consumption

## Abstract

This study analysed the effects of a supervised high‐intensity interval training (HIIT) programme on metabolic flexibility and autonomic function in active postmenopausal women. Twenty women (66.6 ± 6.2 years; 67.9 ± 11.1 kg) completed a 6‐week cycling HIIT intervention consisting of three weekly sessions (20 min/session). Each session included five 1‐min bouts at 170–184% of the power output achieved at maximal carbohydrate oxidation (MCO) determined during a submaximal graded FAT_max_ test, interspersed with 1‐min active recovery at 0.5 W/kg. The initial 2‐week period was included to progressively increase training intensity. Pre‐ and post‐intervention assessments comprised a 30‐min basal metabolic rate evaluation with continuous heart rate variability and gas‐exchange measurements. Paired comparisons with bar and scatter plots were performed. At MCO intensity, significant improvements were observed in MCO (*d* = 0.76), ${\dot{V}}_{O_{2}}$ (*d* = 1.04), gross and net (*d* = 1.63; *d* = 1.35) efficiency, and relative power (*d* = 2.67), with higher Stress Index (*d* = 0.72) and blood lactate (*d* = 0.55). Maximal fat oxidation (MFO) increased from 0.28 to 0.32 g/min (*d* = 0.51) alongside higher sample‐entropy and reduced perceived exertion (*d* = 0.65, *d* = 0.80) at MFO intensity, despite no changes in basal metabolic rate or body composition. Changes in ${\dot{V}}_{O_{2}}$ were negatively correlated with age (*R* ^2^ = 0.34), whereas net and gross efficiency improvements were positively correlated with this latter (*R* ^2^ = 0.23 and 0.26). Six weeks of HIIT elicited safe, time‐efficient metabolic and autonomic adaptations in postmenopausal women. While power output, ${\dot{V}}_{O_{2}}$, carbohydrate oxidation, efficiency and Stress Index improved at MCO intensity, enhanced fat oxidation accompanied by greater autonomic complexity and reduced perceived effort reflect key findings at MFO.

## Abstract (highlights)

- **What is the central question of the study?**
  Does short‐term high‐intensity interval training elicit metabolic and autonomic adaptations during exercise, particularly at maximal fat oxidation and maximal carbohydrate oxidation intensities, in active postmenopausal women.
- **What is the main finding and its importance?**
  High‐intensity interval training improved substrate oxidation, oxygen consumption, efficiency and sympathetic activation, with the most relevant adaptations at the intensity at which MFO was reached (FAT_max_), where increased fat oxidation was accompanied by greater autonomic complexity and lower perceived effort, highlighting integrative autonomic–metabolic adaptations.

## 1 INTRODUCTION

Ageing is linked with progressive loss of muscle mass and function, reducing independence and quality of life (Grosicki et al., 2022). An early distinctive hallmark of this process is an impairment in mitochondrial dynamics and mitochondrial respiration (Bishop et al., 2024), which compromises oxidative capacity and maximal oxygen uptake and contributes to age‐related declines in functional performance (Goulding et al., 2024). Women may exhibit a steeper decline than men, partly due to hormonal changes after menopause (Goulding et al., 2024). Mitochondrial dysfunction can compromise ATP production (Bishop et al., 2024), impairing fat oxidation capacity and thereby promoting metabolic inflexibility, which is closely associated with the development of obesity and type 2 diabetes (Frandsen et al., 2020).

Recent evidence suggests that non‐linear heart rate variability (HRV) metrics may capture early metabolic alterations (Zamora‐Justo et al., 2025), potentially reflecting this metabolic inflexibility from a cardiac autonomic perspective. Age‐related autonomic dysfunction, characterized by reduced vagal activity, may further impair lipid mobilization in adipose tissue (Sun et al., 2023) and attenuate hepatic modulation of glucose metabolism (Brito et al., 2025), potentially contributing to reduced fat oxidation. Indeed, resting sample entropy (SampEn) has recently been related to higher maximal fat oxidation (MFO) during exercise in postmenopausal older women (Monferrer‐Marín et al., 2024). HRV has been proposed as a cost‐effective non‐invasive biomarker of cardiovascular health because of its ability to predict cardiac and autonomic control impairment (Castiglioni et al., 2022).

In this context, regular physical training improves metabolic health through adaptations in mitochondrial content and maximal oxygen uptake (Mølmen et al., 2024) and autonomic regulation (Nascimento‐Carvalho et al., 2024). Exercise intensity determines substrate dynamics, with fat oxidation predominance at low‐to‐moderate intensities, where it reaches its peak rate (MFO) around the 40–45% ${\dot{V}}_{O_{2}\max}$ in sedentary or middle‐aged populations (Amaro‐Gahete et al., 2018). It is noteworthy that it varies markedly with regard to training status, metabolic health and sex (Maunder et al., 2018). Accordingly, training‐induced adaptations may differ across metabolic intensity domains, and types of exercise.

In the last decades, high‐intensity interval training (HIIT) has been shown to be a safe and time‐efficient training method that focuses primarily on intensities close to the maximal carbohydrate oxidation (MCO), improving maximum oxygen consumption (Mølmen et al., 2024) and neuromuscular performance (Hung et al., 2025), even in metabolically inflexible populations such as type 2 diabetics (Hansen et al., 2025) or older adults (Chrøis et al., 2020). However, HIIT‐induced adaptations may not be uniform across all metabolic zones, and other improvements, such as mitochondrial content (Larsen et al., 2015), could impact at lower intensities such as MFO. This remains unknown and may be particularly relevant in the context of ageing, since it is associated with a reduction in the ability to improve fat oxidation at MFO intensities (Frandsen et al., 2020). Furthermore, HIIT has been shown to enhance autonomic regulation, eliciting greater increases in vagal‐related HRV indices than moderate‐intensity exercise at high intensities (Su et al., 2024). However, its effects on autonomic and metabolic responses at intensities near FAT_max_ also remain unclear, with no knowledge in female older populations.

Given the intensity‐dependent heterogeneity of adaptations induced by HIIT, responses across the metabolic intensity spectrum remain poorly understood, particularly in older populations. To date, no studies have examined whether HIIT induces domain‐specific metabolic and autonomic adaptations across MFO and MCO intensities in older women. Therefore, the primary aim of this study was to examine the effects of a 6‐week supervised HIIT intervention on metabolic flexibility and cardiac autonomic regulation in active postmenopausal women. Specifically, we evaluated training‐induced adaptations on energy substrates and vagal‐related indexes at distinct metabolic intensity zones corresponding to MFO and MCO. We hypothesized that HIIT would preferentially enhance performance and carbohydrate oxidation at MCO, whereas changes in fat oxidation would be smaller in magnitude. Additionally, we hypothesized that HIIT would enhance autonomic responsiveness to exercise, reflected by a higher sympathetic activation at intensities close to the second ventilatory threshold (VT2) to allow larger and more efficient responses to vigorous exercise. This would not imply a chronic increase in sympathetic tone, but rather an improved capacity to dynamically regulate autonomic output according to metabolic demand.

## 2 METHODS

### 2.1 Ethical approval

The study followed the ethical standards of the *Declaration of Helsinki*. It was approved by the Ethics Committee of the University of Valencia (reference: 2024‐FIS‐3251696). Before participation, all participants signed a written informed consent form after being fully informed of the aims of the studies and the possible side effects of the procedures.

### 2.2 General design and participants

This investigation followed a single‐arm quasi‐experimental design with pre‐ and post‐intervention measurements to analyse the effects of a short HIIT intervention. Twenty active women volunteered to participate, and completed 8 weeks of training of which the last 6 weeks corresponded to a HIIT protocol, part of the POWER Health study registered as a clinical trial (NCT06336070). A *post hoc* power analysis with the G*Power software (version 3.1.9.6; Heinrich‐Heine‐Universität Düsseldorf, Düsseldorf, Germany) demonstrated that a sample size of *n* = 20 would provide the target statistical power of 0.75 to detect a medium effect size (*d*_z_ = 0.55) with Student's one‐tailed paired‐samples *t*‐test at an α‐level of 0.05 for the MFO rate, as the primary outcome.

Inclusion criteria were: (1) women over 60 years of age, (2) >12 consecutive months of amenorrhoea, (3) moderately active (over 600 METS/week) according to the International Physical Activity Questionnaire (IPAQ), and (4) no medical contraindications to physical exercise. Exclusion criteria included: (1) diagnosed with chronic disease, (2) use of medications (e.g., beta‐blockers) that limit and/or affect physical activity, (3) undergoing hormone replacement therapy or any oestrogen treatment, and (4) episodes of hypotensive response to exercise.

### 2.3 Experimental procedures and assessments

**FIGURE 1:**
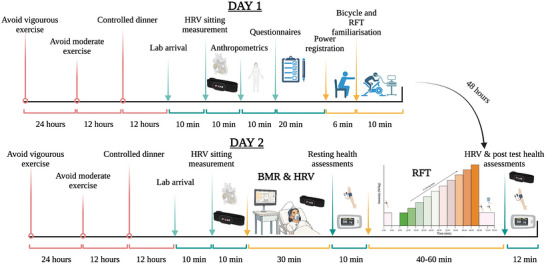
Testing procedures. BMR, basal metabolic rate; BP, blood pressure; HRV, heart rate variability; Power, power values in the five sit to stand test (5STS); RFT, relative to body mass FAT_max_ test‐for further calculation of metabolic flexibility and the maximum intensity of carbohydrate oxidation.

All women underwent 2 days of assessment, with at least 48 h of rest in between (Figure 1), followed by four sessions of familiarization with the cycle ergometer and HIIT protocol during the first 2 weeks, and then 6 weeks of HIIT training, with three sessions per week, as described in the next section. Participants were recreationally active (600–3000 METS/week) and were instructed to maintain their habitual physical activity levels throughout the intervention period, apart from the supervised training sessions.

Before and after the 8‐week intervention (six of HIIT), the 2 days of assessment were repeated to analyse the responses to HIIT intervention. These evaluations were conducted after an overnight fast, where the content of the evening meal prior to testing was standardized to ensure a total energy intake of over 900 kJ, including a minimum of 550 kJ from carbohydrates. Participants were instructed to maintain their habitual dietary patterns throughout the intervention period and to avoid substantial changes in nutritional intake. Additionally, before the post interventions assessment, the participants were asked to refrain from moderate‐to‐vigorous physical activity during the 48 h preceding the pre‐ and post‐intervention testing sessions in order to minimize the influence of changes in exercise and dietary variability on metabolic and autonomic measurements. All assessments were conducted in the same laboratory, using identical equipment, protocols and evaluators at pre‐ and post‐intervention, thereby reducing technical and procedural variability. Testing sessions were scheduled at the same time of day for each participant, and all measurements were performed under strictly controlled thermoneutral conditions (20–23°C), with controlled noise exposure and personnel presence, minimizing environmental influences on autonomic regulation and substrate oxidation.

Potential seasonal confounding was minimized by a narrow and homogeneous intervention window, as all participants initiated the programme between late winter and early spring and completed it by late spring or early summer, reducing the likelihood of seasonal effects on key markers. In addition, participants were instructed to maintain their habitual physical activity levels outside the intervention, which were monitored throughout the study and showed no systematic changes, limiting the influence of external training adaptations.

At the first test day, participants arrived at the lab and, after a 10‐min rest, heart rate (HR), arterial oxygen saturation ($S_{aO_{2}}$) was recorded with a Nonin Onyx Vantage 9590 pulse oximeter (Nonin Medical, Plymouth, MN, USA). Blood pressure (BP) was measured three times, once before and twice after the test, once during the 5‐min passive recovery on the bicycle and once at the end of this period, at rest, using an Omron M6 sphygmomanometer (Omron Healthcare, Kyoto, Japan). Height was measured (SECA 222, Seca GmbH, Hamburg, Germany), body composition assessed via bioelectrical impedance (Tanita DC‐430 MA S, Tanita Corporation, Tokyo, Japan), and relative muscle power (W/kg) evaluated with the five‐repetition sit‐to‐stand test (5STS) (Alcazar et al., 2018). Finally, participants completed a familiarization session on the cycle ergometer (Saris H3, Saris, Madison, USA), during which participants were instructed and practiced maintaining their self‐preferred cadence, always within the predefined range (55–85 rpm) to ensure comfort and power compliance during the subsequent incremental test.

Gas analyses were performed on the second assessment day using the Cosmed K5 metabolic cart (Cosmed, Rome, Italy, Figure 1). Before recording, participants arrived in the morning (08.30 or 10.00 h), having avoided strenuous physical activity for the previous 48 h and moderate physical activity for the last 24 h. After 15 min of rest in the seated position, recording of basal metabolic rate (BMR) was performed for 30 min in the supine position. Subsequently, participants performed a 4‐min warm‐up on the cycle ergometer at 0.45 W/kg, followed by 4 min rest and then the FAT_max_ protocol was started. The incremental test was adjusted for body weight (RFT), starting at 0.45 W/kg and increasing by 0.15 W/kg every 4 min.

The protocol was designed to identify the intensities at which fat oxidation (MFO) and carbohydrate oxidation (MCO) rates peaked. ${\dot{V}}_{O_{2}}$ or ${\dot{V}}_{CO_{2}}$ was continuously recorded to calculate fat and carbohydrate rates, respiratory exchange ratio (RER), as well as respiratory frequency. It is noteworthy that when women slowed (<50 rpm) or increased cadence over their usual (>80 rpm), the researcher helped them to adjust power to their comfortable cadence by means of a Grip Shift. Since the protocol was not designed to determine ${\dot{V}}_{O_{2}\max}$, no maximal criteria (e.g., cadence <50 rev/min or volitional exhaustion) were needed as endpoints.

MFO was defined as the intensity eliciting the highest fat oxidation. MCO corresponded to the final stage of the RFT test, defined as the point where the RER exceeded 1.0. If this intensity was not achieved, the test was terminated when participants were unable to maintain the power required, as indicated by a rating on the rate of perceived exertion (RPE) scale greater than 6 (0–10) or a score on the visual analogue scale (VAS) for pain greater than 5 (0–10). During all exercise assessments and training sessions, cycling posture was strictly standardized. Participants remained seated throughout the protocol, with both hands always placed on the handlebars, avoiding changes in trunk position or upper‐body support that could influence mechanical efficiency, ventilation or autonomic responses. Cadence was continuously monitored and verbally reinforced by the evaluators to remain within a controlled range, ensuring that it did not fall below 55 rpm or exceed 85 rpm.

Later, gross efficiency (GE) and net efficiency (NE) were calculated. These variables were complemented with blood lactate measurements obtained both before starting the incremental test and 3 mins after its completion, using capillary samples collected from the index finger of the left hand (Lactate Scout Sport Solo device, SensLab GmbH, Berleben, Germany).

On day 2, HRV was monitored during both the 30‐min supine BMR recording and the RFT protocol using the Polar H10 chest strap device (Polar Electro Oy, Kempele, Finland). Subsequently, RR recordings were exported from the Polar Sensor Logger app to Kubios Scientific software (version 4.0.2; Biosignal Analysis and Medical Imaging Group, Department of Physics, University of Kuopio, Kuopio, Finland) for further analysis.

### 2.4 HIIT intervention

**FIGURE 2:**
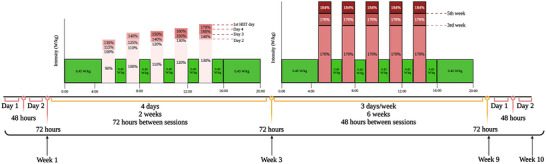
Study protocol illustrating the training stages (familiarization and HIIT sessions) over time, with intensity in % of power in W/kg at maximum carbohydrate oxidation (MCO) intensity.

Training started with a 2‐week familiarization period, that consisted of four sessions maintaining the HIIT format of five 1‐min bouts interspersed with 1‐min active recovery at 0.45 W/kg (Figure 2). This familiarization phase allowed participants to adapt to the ergometer, interval structure, perceptual demands and workload prescription, thereby stabilizing neuromuscular and autonomic responses prior to the intervention phase. Exercise intensity, expressed as a percentage of the MCO power determined in the RFT test, was progressively increased both within and across sessions. This approach was selected due to its demonstrated reproducibility in the studied population (Søgaard et al., 2018).

In sessions 1 and 2, participants started at 90% and finished at 130% and 140% of MCO power, respectively. In sessions 3 and 4, intensity started at 100% and progressed to 160% and 170%, corresponding to the levels prescribed for the initial weeks of the HIIT intervention as reflected the Figure 2. The training protocol was adapted from Søgaard et al. (2018).

This approach was selected because MCO occurs below ${\dot{V}}_{O_{2}\max}$ and maximal external workload (*W*_max_), typically close to the second ventilatory threshold, as indicated by average RER values <1. Prescribing intensity in relation to the MCO provides an individualized reference workload, whilst avoiding reaching maximum intensities during the incremental test used to determine training zones. Moreover, the short duration of the work intervals (1 min) enables participants to tolerate workloads above the steady‐state domain without excessive fatigue, a strategy commonly used in HIIT protocols for older adults (Whitehurst, 2012). The progressive increase in workload during the familiarization phase further ensured a gradual and safe transition to the target training intensities.

After the 2 weeks of familiarization, all women completed 6 weeks of supervised HIIT on a cycle ergometer training 3 times per week. The structure of five 1‐min bouts per session, the weekly volume and intervention duration followed the design of previous studies (Chrøis et al., 2020; Søgaard et al., 2018). Each HIIT session comprised a 5‐min warm‐up at 0.50 W per kilogram of body weight, followed by five 1‐min HIIT intervals at an individually determined workload (cadence >45 rpm). This workload corresponded to 170% of MCO power in the RFT protocol and was implemented following the four familiarization sessions, continuing until completion of the first six sessions or the initial 2 weeks of the HIIT training period. The intervals were interspersed with 1‐min periods of cycling at 0.50 W/kg or complete rest on the cycle ergometer, followed by a 5‐min cool‐down at 0.50 W/kg after the five sets.

At the beginning of the third and fifth weeks of the HIIT block, that is, sessions 6 and 12, and contingent upon objective (HR) and perceptual (RPE) indicators showing reduced strain in the preceding sessions, the relative power of the work sets was recalculated, with increases of up to 176% and 184% of the power at MCO intensity in weeks 3 and 5, respectively, to maintain the target stimulus until the end of the intervention (Figure 2).

Adherence to the training programme was complete, as additional sessions were individually scheduled when necessary to ensure 100% attendance across the 18 planned sessions. To characterize internal and external training load during the HIIT intervals, HR and rating of perceived exertion (RPE) were recorded as internal load indicators, while relative power output (RelPower) represented external load. For descriptive purposes, these variables were analysed in the final session of each training block, which represent the progressive stages of the HIIT intervention.

All the sessions were conducted under postprandial conditions and in the same laboratory environment used for testing, with only one or two participants training simultaneously, allowing close supervision and individualized load adjustment. The Polar H10 chest strap device was used to continuously monitor HR and HRV during all sessions, thereby verifying internal load consistency and participant safety across sessions. In addition, oxygen saturation levels were measured for security (Onyx Vantage fingertip pulse oximeter; Nonin Medical). At the conclusion of each high intensity interval, exertion and pain scales were assessed. The Rouvy AR application was used to configure all intensity levels, which were then transferred to the Saris H3 smart roller (CycleOps Hammer Direct Drive Trainer, Saris, Madison, WI, USA), ensuring precise and reproducible workload delivery across all sessions and participants.

### 2.5 Calculations

The relative muscle power calculations (RelPower 5STS) were assessed with the 5STS test, performed twice with 60 s rest following Alcazar et al. (2018), using a standardized armless chair (0.47 m) and timed to the nearest 0.01 s. Relative muscle power (W/kg) was calculated as the ratio of STS muscle power to body mass.

BMR (in kilocalories per day) was estimated using the equation proposed by Weir (1949). For the analysis, the first 5 min was discarded, given the transient variations in oxygen consumption and CO_2_ production in this period that may alter the accuracy of the calculation (Fullmer et al., 2015).

Metabolic flexibility calculations were based on the gas analysis windows of the first 60 s of the last 90 s of each intensity, that is, discarding the last 30 s. Using the equation of Frayn (1983), the MFO point, reached at the FAT_max_ intensity, and the maximal carbohydrate point (MCO), reached at the end of the test, were determined.

GE was calculated as the ratio between the external mechanical work performed at a given intensity and its metabolic cost (Blasco‐Lafarga et al., 2022):

GE (%) = (Power (W)/3.90${\dot{V}}_{O_{2}}$ (l/min) + 1.10 ${\dot{V}}_{CO_{2}}$ (l/min) × 69.7) × 100

Net efficiency (NE) was calculated by subtracting BMR from total energy expenditure (EE), thereby reflecting only the additional energy cost attributable to exercise (Broskey et al., 2015):

NE (%) = {Power (W)/[(EE (kcal/min) – EE_rest_ (kcal/min)] × 69.7} × 100

Both NE and GE, like the energy substrates, were analysed in windows of the first 60 s of the last 90 s of each intensity, again discarding the last 30 s.

For the HRV analysis with Kubios software, artefacts were identified and corrected (lambda = 500) using the Kubios ‘automatic method’, and those exceeding 2% in the baseline recording, and 3% in the RFT protocol recording, were excluded from the analysis following previous studies (Blasco‐Lafarga et al., 2017; Monferrer‐Marín et al., 2024). HRV at MFO and MCO intensities was retained for further analysis.

Specifically, the linear variables RMSSD and Baevsky's Stress Index were calculated in 60‐s windows at the end of the stage, after discarding the last 30 s, in line with metabolic flexibility window analysis, because these variables guarantee sufficient stability (Shaffer & Ginsberg, 2017). The root mean square of successive normal beat‐to‐beat differences (RMSSD, in milliseconds) was selected as the primary linear variable, as it reflects vagal reactivation (Laborde et al., 2024). The Stress Index reflects cardiovascular stress, with high values indicating significant sympathetic activation and reduced variability (Blasco‐Lafarga et al., 2017). In contrast, the non‐linear variables (SampEn, detrended fluctuation analysis alpha‐1 (DFAα1)) and time domain power variables were analysed in 120‐s windows, excluding the final 30 s of each intensity, as these measures require longer recording periods (Blasco‐Lafarga et al., 2017; Shaffer & Ginsberg, 2017). Artefact rates were quantified for each recording using Kubios HRV software as the percentage of corrected RR intervals relative to the total number of beats, and were summarized as the mean ± SD at each assessment (PRE and POST).

### 2.6 Statistical analysis

All statistical analyses and figure generation were performed using Python 3.13.3. (Python Software Foundation, Wilmington, DE, USA). Normality of the data was assessed using the Shapiro–Wilk test. Depending on the distribution, paired sample comparisons were conducted using either Student's *t*‐test or Wilcoxon's signed‐rank test. These analyses were applied to variables related to body composition, BMR and muscular power (Table 1), as well as to key metabolic variables at MFO and MCO (Figure 2). Bar plots were used to visually represent the most relevant variables for each intensity domain. Additionally, scatter plots were generated to evaluate the changes induced by the intervention and the influence of age. The deltas, defined as the difference between POST and PRE values, were calculated for efficiency variables, carbohydrate oxidation rate, blood lactate and ${\dot{V}}_{O_{2}}$ at MCO, as well as fat oxidation rate and SampEn at MFO, and relative power at both intensities (Figure 4). These delta values were used to visually explore associations between training‐induced changes across variables and their relationship with age.

**TABLE 1:** Subject characteristics before and after 6 weeks of HIIT.

Variable	PRE	POST	*P*	Effect size
Age (years)	66.6 ± 6.2			
Weight (kg)	67.9 ± 11.1	67.6 ± 11.1	0.226	0.28
Fat mass (kg)	23.9 ± 7.7	23.4 ± 7.8	0.060	0.47
LM (kg)	41.7 ± 4.4	42.0 ± 4.5	0.157	0.32
SBP (mmHg)	124 ± 16	125 ± 18	0.558	0.13
DBP (mmHg)	78 ± 7	77.7 ± 8	0.774	0.07
HR (bpm)	64.5 ± 7.2	64.9 ± 13.2	0.245	0.27
BMR (kcal/day)	1012 ± 250	968 ± 299	0.347	0.21
RER	0.79 ± 0.05	0.80 ± 0.06	0.342	0.22
RelPower 5STS (W/kg)	3.30 ± 0.70	3.65 ± 0.70	<0.001	1.11
Baseline lactate (mmol/l)	1.41 ± 0.64	1.35 ± 0.73	0.764	0.07

Data are presented as means ± SD (*n* = 20). Abbreviations: BMR, basal metabolic rate; DBP, diastolic blood pressure; HR, heart rate; LM, lean mass; RelPower 5STS, relative power in five times sit‐to‐stand test; RER, respiratory exchange ratio; SBP, systolic blood pressure.

## 3 RESULTS

### 3.1 Participant characteristics

The 6‐weeks HIIT intervention was divided into three blocks with main load variables averaged as follows. Session 6 (final session of the first training block; at the end of week 3) was performed at 170% of MCO power, with HR of 128 ± 12 bpm, RPE of 5.6 ± 0.7 in Borg 0–10 scale; and 1.9 ± 0.6 W/kg. Session 12 (final session on the second training block, end of week 5), was performed at 177% of MCO power (139 ± 11 bpm, 5.1 ± 1.0 RPE, 1.94 ± 0.63 W/kg). At the end of the intervention, session 18 (end of the third training block, week 6), the training was performed at 184% of MCO power and averaged 148 ± 13 bpm and 4.0 ± 2.1 RPE at 2.02 ± 0.66 W/kg.

After this period, no changes were found in body composition (Table 1). Only fat mass showed a trend with a moderate effect size of loss (*d* = 0.47) after the intervention. No changes were observed for BMR RER or basal blood lactate. However, muscle power measured with the 5STS protocol showed a significant improvement, with a large effect size (*d* = 1.11).

### 3.2 HRV

The average artefact rate during the FAT_max_ test before the intervention was 0.21 ± 0.26%, and 0.30 ± 0.48% after the HIIT training period, remaining below the predefined exclusion thresholds in all participants included.

### 3.3 HIIT changes at MFO and MCO intensities

**FIGURE 3:**
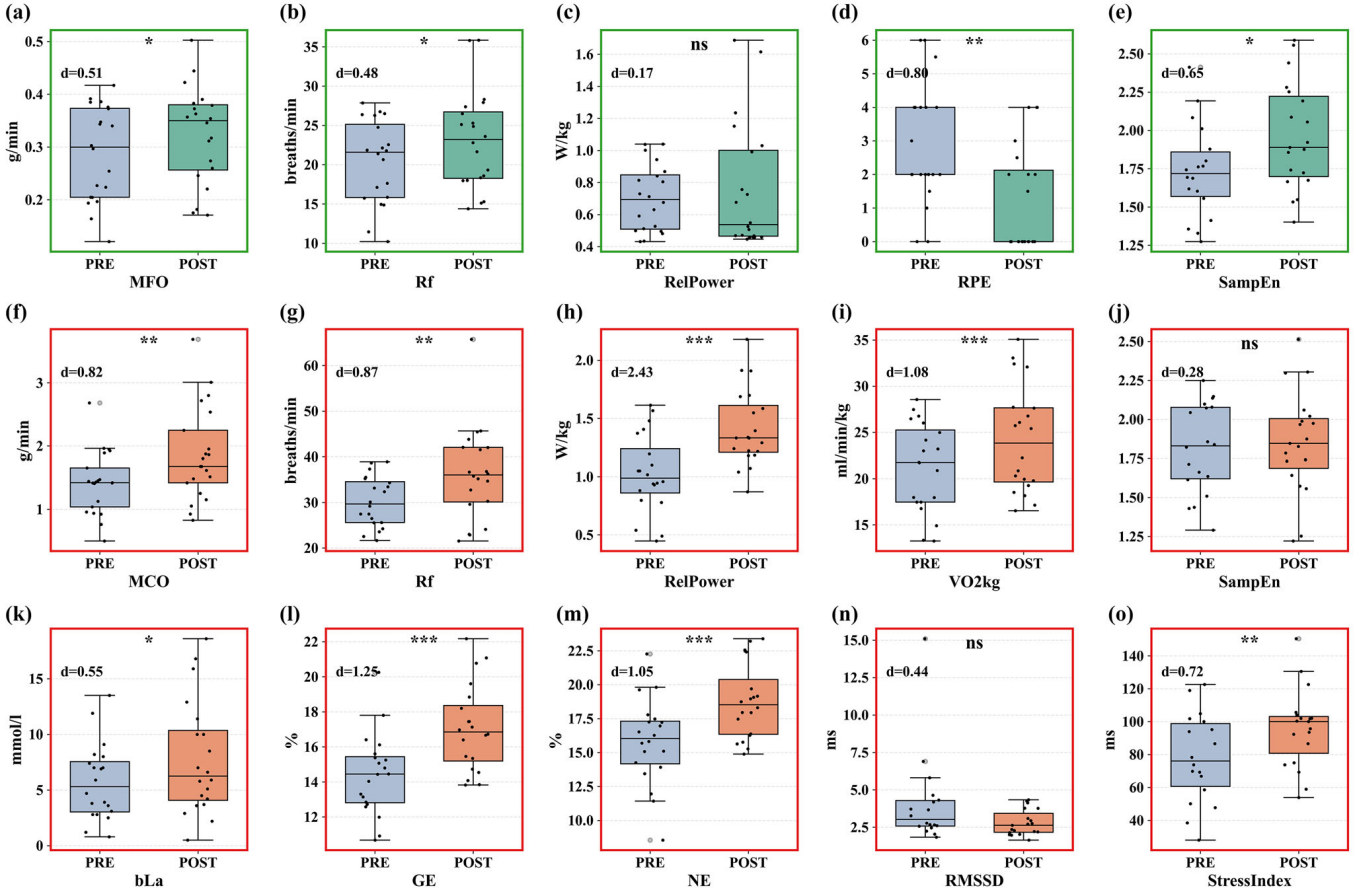
Boxplot of HIIT effects on MFO and MCO intensities (*n* = 20). (a–e) Variables at FAT_max_ intensity (MFO): maximal fat oxidation rate (MFO, a), respiratory frequency (Rf, b), relative power (RelPower, c), perceived exertion (RPE, d) and sample entropy (SampEn, e). (f–o) Variables at the exercise intensity of maximal carbohydrate oxidation (MCO): MCO (f), respiratory frequency (g), relative power (h), ${\dot{V}}_{O_{2}}$ relative to body mass (VO2kg, i), sample entropy (j), blood lactate (3 min after MCO, bLa, k), gross efficiency (GE, l), net efficiency (NE, m), root mean square of successive differences of beats (RMSSD, n), and stress index (o). *P < 0.05, **P < 0.01, ***P < 0.001.

After 6 weeks of HIIT, at MCO intensity relative power (*d* = 2.43), net efficiency (*d* = 1.26) and GE (*d* = 1.05) increased significantly. Maximal oxygen consumption (*d* = 1.08), respiratory rate (*d* = 0.87), blood lactate (*d* = 0.55) and MCO rate also increased (*d* = 0.80). The Stress Index increased (*d* = 0.72) and RMSSD showed a small decrease (*d* = 0.44, *P* = 0.091). At MFO intensity, MFO rate increased from 0.28 ± 0.09 to 0.32 ± 0.09 g/min (*d* = 0.51), simultaneously with a decrease in perceived exertion (*d* = 0.80), and increased respiratory frequency and sample entropy (*d* = 0.48 and 0.65, respectively) (Figure 3).

### 3.4 Age and HIIT changes relationships

**FIGURE 4:**
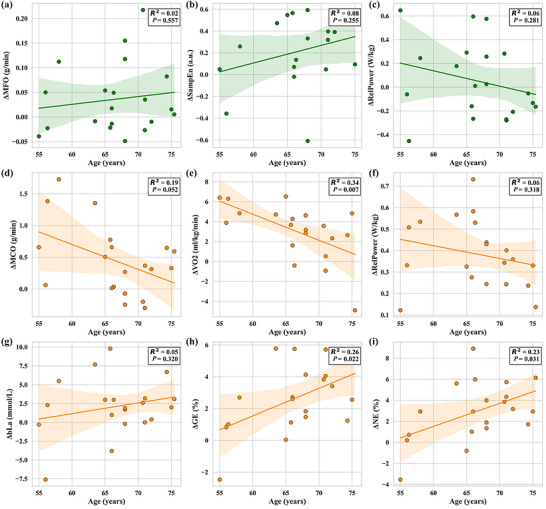
Scatter plots with 95% confidence intervals shading showing the effect of age on Δ changes after HIIT (*n* = 20). (a–c) Variables at MFO: MFO rate (a), sample entropy (b), and relative power (c). (d–i) Variables at MCO: MCO rate (d), maximal oxygen consumption (${\dot{V}}_{O_{2}}$, e), relative power (f), blood lactate (g), gross efficiency (GE, h), and net efficiency (NE, i).

The relationships between age and the changes induced by HIIT (expressed as delta values, post–pre) are illustrated in Figure 4. At MCO intensity, Δ${\dot{V}}_{O_{2}}$ showed a significant moderate negative correlation with age, indicating smaller ${\dot{V}}_{O_{2}}$ improvements in older participants. A similar, near‐significant trend was observed for ΔMCO. No clear associations with age were found for ΔRelPower or ΔbLa. In contrast, both ΔGE and ΔNE showed significant positive correlations with age, suggesting relatively larger efficiency gains in older participants. At MFO intensity, no associations with age were observed.

## 4 DISCUSSION

This study provides novel evidence that a brief HIIT intervention, just a volume of 60 min per week, for 6 weeks, induces significant improvements in MFO intensity in active postmenopausal women over 60, with direct implications for their health. At MFO intensity, HIIT enhanced fat oxidation, increased cardiac signal complexity (sample entropy) and reduced perceived exertion. These changes are consistent with concurrent improvement in autonomic regulation and metabolic flexibility, which may support better exercise tolerance in this population. At MCO intensity, improvements extend beyond mechanical output and oxygen consumption to include greater metabolic efficiency (GE and NE), enhanced carbohydrate oxidation and blood lactate, accompanied by an increased Stress Index and breathing frequency. These autonomic and metabolic improvements highlight HIIT as a safe and time‐efficient intervention also in these women and underscore the value of HRV as a sensitive biomarker of metabolic and cardiovascular adaptations to training.

Body composition remained largely unchanged following the intervention. Fat mass showed a small, non‐significant reduction (−0.50 ± 0.24 kg, *P* = 0.06), a trend comparable to that reported by Søgaard et al. (2018), who observed modest changes in fat mass after HIIT in a similar cohort. Although this tendency should be interpreted with caution, it is noteworthy in the context of menopause, a condition commonly associated with reduced fat oxidation capacity (Maillard et al., 2018). The concurrent increases in Stress Index, and breathing frequency observed during exercise reflect greater autonomic and ventilatory engagement at higher intensities (i.e., sympathetic response), which may be compatible with transient increases in post‐exercise lipid utilization, although it cannot be directly linked to changes in fat mass with the present single‐arm design. The greater heterogeneity of the variable around the test, especially in intensities below VT2, may explain these data (Fleitas‐Paniagua et al., 2023).

It is noteworthy that no changes were observed in resting metabolic parameters (BMR, RER and basal lactate), suggesting that the HIIT stimulus was insufficient to significantly alter basal energy metabolism. This is consistent with previous interventions in younger (Garcia‐Romero et al., 2023) and older (Søgaard et al., 2018) populations, and may also indicate that, similar to body composition, longer training periods are required to elicit adaptations, taking into account that ageing may slow down the process of muscle hypertrophy (Endo et al., 2020).

Among the intensities tested, the greater adaptations to HIIT were observed at the MCO threshold. This was determined based on the greatest effect sizes for relative power, oxygen consumption, metabolic efficiency and carbohydrate oxidation rate, as hypothesized. HIIT has been associated with peripheral adaptations such as faster ${\dot{V}}_{O_{2}}$ kinetics and greater O_2_ extraction and utilization, particularly at higher intensities near VT2 (Liu et al., 2023). In the present study, the interval workload corresponds to 120% of *W*_max_ in the first training block, reaching 130% of *W*_max_ in the third one. Active older women improved their carbohydrate oxidation capacity (∼0.45 g/min; >30%) and blood lactate production (>2 mmol/l) after the HIIT intervention in MCO intensity. These responses may reflect enhanced glycolytic capacity, potentially supported by greater sympathetic response (Monferrer‐Marín et al., 2024), also in our study (i.e., larger Stress Index at MCO). Such adaptations are consistent with previous evidence showing that HIIT upregulates glycolytic enzymes and enhances lactate transporters (Abe et al., 2015). In contrast, previous studies in trained individuals have reported reduced lactate responses following interval training (Astorino et al., 2022). The discrepancy may be explained by the higher workloads after our intervention, with relative power output increasing by 0.38 W/kg (∼37%; *d* = 2.43). This greater external load was accompanied by a higher internal load, reflected by an increase in HR from 136 to 143 bpm at MCO.

Maximal oxygen consumption increased by 3 mL/min/kg after only 6 weeks of HIIT (three weekly, 5 min per session, high intensities). This increase is consistent with ${\dot{V}}_{O_{2}}$ adaptations previously reported after HIIT in younger obese populations and older adults (Oliveira et al., 2024), and somewhat lower than those observed in healthy adults (Wen et al., 2019). This reduced response may be related to the limited capacity to further increase cardiac output with advancing age (Mølmen et al., 2024). Nevertheless, HIIT has been shown to elicit greater improvements in cardiorespiratory fitness, compared to continuous moderate intensities, in older populations (Poon et al., 2021), highlighting its potential as a time‐efficient strategy to improve aerobic capacity. Such adaptations are particularly relevant for postmenopausal women, given the age‐related decline (Valenzuela et al., 2020) and menopause hormonal changes (MacGregor et al., 2025). Additionally, ${\dot{V}}_{O_{2}\max}$ remains a strong predictor of all‐cause mortality risk (Fung et al., 2021).

A similar pattern emerged for carbohydrate oxidation, consistent with murine studies reporting age‐related declines in glycolytic capacity (Karaś et al., 2024), whereas no differences were observed in fat oxidation. In contrast, gross and net efficiency showed greater relative improvements in the older cohort, allowing them to reach values comparable to the younger cohort despite lower baseline levels. This pattern may indicate adaptive responses that help to preserve the benefits of HIIT in aged subjects.

Maximal power output exhibited the most substantial enhancement following the intervention, increasing by 40%, which enabled participants to produce an additional 25 W on average and extend their performance duration in the RFT protocol by approximately 12 min. These adaptations may influence the improvement in MFO, as recent evidence suggests that in this population muscle power limitations can shorten incremental test duration and markedly reduce time spent in the oxidative zone (Blasco‐Lafarga et al., 2022). Therefore, these muscle power improvements may be related to greater oxygen extraction at the muscular and mitochondrial levels, as previously suggested following HIIT interventions (Lundby & Jacobs, 2016), potentially facilitating greater lipid substrate utilization at MFO intensity. Functional improvements were also reflected in increased muscle power measured by the 5STS test, which has been associated with reduced fall risk and all‐cause mortality (Goulding et al., 2024). Enhanced muscle performance following the 6‐week HIIT intervention may therefore contribute to functional capacity and autonomy in older adults, even without measurable changes in muscle mass (Goulding et al., 2024). Of the utmost importance, these interpretations should be considered cautiously, as mitochondrial adaptations and fall risk were not directly assessed in the present study.

Unexpectedly, at the MFO intensity, postmenopausal women increased their fat oxidation capacity without changes in oxygen uptake or mechanical output. However, a clear reduction in perceived exertion and a greater sample entropy were observed. This increase in MFO rate is particularly noteworthy as previous studies have reported limited or inconsistent effects on fat oxidation and mitochondrial function, often identifying changes in mitochondrial content rather than oxidative capacity as the primary adaptation (Chrøis et al., 2020; Larsen et al., 2015). HIIT would thus improve lipid mobilization and utilization, as previously suggested (Atakan et al., 2022), although the underlying mechanisms cannot be directly inferred from the current data. This increase in MFO occurred alongside a reduction in perceived exertion and a concomitant rise in sample entropy, suggesting an enhanced capacity of the autonomic nervous system to dynamically respond to physiological demands through improved coordination between sympathetic and parasympathetic activity (Chua et al., 2008). At a given exercise intensity, autonomic regulation might operate more efficiently, requiring less perceptual effort to maintain homeostasis, as suggested by previous literature (Bönhof et al., 2022).

Notably, SampEn increased in parallel with fat oxidation, supporting the interpretation of SampEn as a marker related to global autonomic complexity rather than a purely vagal or linear signal. This interpretation is consistent with evidence identifying SampEn as an early indicator of metabolic dysregulation (Zamora‐Justo et al., 2025) and with previous observations linking higher resting SampEn to greater MFO during exercise (Monferrer‐Marín et al., 2024), and also in resting conditions (Monferrer‐Marín et al., 2026). Although speculative, this parallel behaviour may reflect a tighter integration between autonomic regulation and substrate utilization during exercise following HIIT, potentially consistent with previously described vagal influences on systemic metabolic regulation (Imai & Katagiri, 2022).

To summarize, the present study supports the inclusion of nonlinear HRV metrics in the investigation of exercise metabolism, particularly in populations with impaired metabolic flexibility, while emphasizing that the observed associations should be interpreted as functional adaptations. Collectively, HIIT can elicit concurrent improvements in fat oxidation capacity and autonomic regulation at MFO intensity in active postmenopausal women. Within the participants, age influenced the response to HIIT: lower improvements in oxygen consumption with increasing age, but greater gains in mechanical efficiency. Although these results are encouraging, they should be interpreted with caution given the single‐arm design. Since the study included no control group and was conducted in a population commonly characterized by age‐related impairment in mechanical and metabolic function (Frandsen et al., 2020), its adaptations should be interpreted cautiously and cannot be directly attributed to the intervention beyond descriptive associations.

### 4.1 Study limitations

Nevertheless, some limitations should be acknowledged. First, we did not include a control group and/or an endurance training group for direct comparison, and this restricts causal inference and limits the generalizability of the study. However, all control variables have been specified in order to avoid internal validity weaknesses. Second, the analysis focused on two key workloads, chosen to capture the most relevant physiological responses. Studies with more subjects are warranted to support and confirm our hypotheses, as well as further areas of analyses that allow observation of how HIIT responses affect any intensity. Moreover, other cohorts of inactive older adults or individuals with impaired metabolic flexibility, who may exhibit different physiological responses to HIIT, may determine the broader applicability of these adaptations. Additionally, substrate oxidation measurements may exhibit some day‐to‐day variability. Although MFO has shown no systematic bias across repeated assessments, some intra‐individual variability is expected regardless of the analytical method employed (Chrzanowski‐Smith et al., 2020). Finally, while the sample size was sufficient to detect within‐subject changes, larger cohorts and repeated FAT_max_ assessments would help to confirm these findings and better characterize inter‐ and intra‐individual variability in training responses across the full intensity spectrum.

### 4.2 Conclusion

Our findings show significant physiological adaptations in physically active postmenopausal women after a 6‐week HIIT programme (20‐min sessions, three times per week). At MFO intensity, women increased their fat oxidation rates without a concomitant shift in the workload corresponding to MFO. Notably, this adaptation was accompanied by higher sample entropy, suggesting concurrent adaptations in HRV complexity and fat oxidation. At MCO intensity (RER ≈ 1), improvements were observed not only in mechanical output and oxygen consumption, but also in carbohydrate oxidation, blood lactate, sympathetic response, and both gross and net efficiency, despite the known age‐related constraints on these variables. Finally, the improvement in relative power (5STS) highlights the effectiveness (both physiological and functional) of this short HIIT intervention, supporting its safety and time effectiveness in postmenopausal women. Taken together, these findings should be interpreted as functional adaptations consistent with improved autonomic–metabolic integration.

## AUTHOR CONTRIBUTIONS

Jordi Monferrer‐Marín: conceptualization; investigation; data curation; methodology; formal analysis; writing – original draft preparation; writing – review and editing. Ainoa Roldán: investigation; methodology; review and editing. Jørn Wulff Helge: writing – review and editing. Cristina Blasco‐Lafarga: conceptualization; project administration; investigation; methodology; formal analysis supervision; writing – original draft preparation; writing – review and editing. All authors have read and approved the final version of this manuscript and agree to be accountable for all aspects of the work in ensuring that questions related to the accuracy or integrity of any part of the work are appropriately investigated and resolved. All persons designated as authors qualify for authorship, and all those who qualify for authorship are listed.

## CONFLICT OF INTEREST

None declared.

## GENERATIVE AI STATEMENT

The authors confirm that no artificial intelligence tools, including large language models (LLMs), were used in the drafting or revision of this manuscript. All content was conceived, written and approved solely by the authors.

## Data Availability

The datasets generated and/or analysed during the current study are not publicly available due to the conditions of the ethical approval provided by the University of Valencia Human Research Ethics Committee. Notwithstanding, the anonymous data and analysis are available from the corresponding author on reasonable request.
